# Design of a New Phthalocyanine-Based Ion-Imprinted Polymer for Selective Lithium Recovery from Desalination Plant Reverse Osmosis Waste

**DOI:** 10.3390/polym15183847

**Published:** 2023-09-21

**Authors:** Bassem Jamoussi, Radhouane Chakroun, Bandar A. Al-Mur, Riyadh F. Halawani, Fahed A. Aloufi, Anis Chaabani, Naif S. Aljohani

**Affiliations:** 1 Department of Environment, Faculty of Environmental Sciences, King Abdulaziz University, Jeddah 21589, Saudi Arabia; rshagroon@kau.edu.sa (R.C.); balmur@kau.edu.sa (B.A.A.-M.); rhalawani@kau.edu.sa (R.F.H.); faloufi@kau.edu.sa (F.A.A.); naljohani3@swcc.gov.sa (N.S.A.); 2 Department of Hydrology and Water Resources Management, Faculty of Environmental Sciences, King Abdulaziz University, Jeddah 21589, Saudi Arabia; achaabani@kau.edu.sa; 3 Saline Water Conversion Corporation, Riyadh 11432, Saudi Arabia

**Keywords:** ion-imprinted polymer (IIP), solid-phase extraction (SPE), Li recovery, desalination plant reverse osmosis waste, central composite design (CCD), response surface methodology (RSM)

## Abstract

In this study, a novel technique is introduced that involves the combination of an ion-imprinted polymer and solid-phase extraction to selectively adsorb lithium ions from reverse osmosis brine. In the process of synthesizing ion-imprinted polymers, phthalocyanine acrylate acted as the functional monomer responsible for lithium chelation. The structural and morphological characteristics of the molecularly imprinted polymers and non-imprinted polymers were assessed using Fourier transform infrared spectroscopy and scanning electron microscopy. The adsorption data for Li on an ion-imprinted polymer showed an excellent fit to the Langmuir isotherm, with a maximum adsorption capacity (Qm) of 3.2 mg·g^−1^. Comprehensive chemical analyses revealed a significant Li concentration with a higher value of 45.36 mg/L. Through the implementation of a central composite design approach, the adsorption and desorption procedures were systematically optimized by varying the pH, temperature, sorbent mass, and elution volume. This systematic approach allowed the identification of the most efficient operating conditions for extracting lithium from seawater reverse osmosis brine using ion-imprinted polymer–solid-phase extraction. The optimum operating conditions for the highest efficiency of adsorbing Li^+^ were determined to be a pH of 8.49 and a temperature of 45.5 °C. The efficiency of ion-imprinted polymer regeneration was evaluated through a cycle of the adsorption–desorption process, which resulted in Li recoveries of up to 80%. The recovery of Li from the spiked brine sample obtained from the desalination plant reverse osmosis waste through the ion-imprinted polymer ranged from 62.8% to 71.53%.

## 1. Introduction

Seawater desalination is an effective method used worldwide for obtaining fresh potable water [1]. However, the environmental effects of seawater reverse osmosis (SWRO) plants have raised several issues. These plants generate large amounts of water and dense saline residues, which are discharged into the marine ecosystem. Lithium is naturally present in seawater with an estimated global reserve of approximately 230 billion tons. However, the concentration is very low, typically around 0.1 to 0.2 parts per million (ppm), which makes the extraction process challenging and expensive. Researchers worldwide are reviewing and updating lithium extraction technologies, including economic and feasibility analyses. Kim et al. outlined the sequence and purpose of various pretreatment steps in lithium-ion battery (LIB) recycling to improve the recovery efficiency of valuable materials and reduce energy consumption [2]. Butt et al. reviewed and compared different methods for lithium extraction and recycling from primary and secondary resources, highlighting the potential of membrane technology as a promising replacement for conventional methods [3]. In 2025, the lithium demand is expected to reach 900,000 metric tons (three times as much as in 2018) [4]. The technology to extract lithium from seawater is still under development, and researchers are exploring various methods to increase the efficiency and cost-effectiveness of the process. Nevertheless, the potential for obtaining lithium from seawater is significant, as it could provide a sustainable source of this critical metal for various industries, including the rapidly growing electric vehicle market [5]. According to recent reports, Japan and South Korea are intending to develop large-scale seawater lithium mining facilities [6,7,8]. A full-scale seawater lithium extraction facility expected to produce 3000 tons of lithium is currently under development in Japan. In South Korea, more than USD 185 million has been invested in similar projects to extract 10,000 tons of lithium from seawater by 2025. With over 32 desalination plants in operation, Saudi Arabia currently produces 3.6 million cubic meters of drinking water every day [9,10]. Researchers at King Abdullah University of Science and Technology (KAUST) have recently developed a new cost-effective method for extracting Li from seawater [11]. If the extraction process could be optimized, the country could potentially enter the global lithium market. However, the commercial viability of this approach is still at the research and development stage, and it faces various technical and economic hurdles that must be addressed before it becomes a reality.

Several approaches have been proposed for the extraction and recovery of lithium from brines. One of the extensively researched methods involves examining the adsorption of lithium onto inorganic materials [12,13,14,15,16] such as zeolites, metal–organic frameworks (MOFs), and layered double hydroxides (LDHs) [17]. In a previous study, we demonstrated the feasibility of extracting lithium from aqueous solutions through the entrapment of lithium ions using a freshly prepared aluminum hydroxide gel in the presence of a strong base [18]. Other separation processes include solvent extraction [19], precipitation, organic resins, polymeric sorbents, and membrane-dependent processes [20,21,22,23]. In recent studies, it has been revealed that the most cost-effective method for lithium recovery is utilizing technology that involves the sorption of lithium using inorganic molecular sieve ion-exchange sorbents [24,25]. However, several challenges remain with the traditional methods for separating lithium ions, such as high energy consumption, inefficient separation, and weak selectivity [26,27]. Due to their energy efficiency and simplicity in continuous operations, membrane separation techniques have become increasingly popular for ion separation [28]. Hoshino et al. have developed a lithium recovery technique that involves impregnating organic membranes with an ionic liquid [29,30]. While this method can be employed to extract lithium from seawater, the unstable and short-lived nature of the ionic membrane presents a significant obstacle to achieving a stable and long-lasting recovery of lithium from seawater. Other technologies currently in development may provide a direct and selective lithium extraction method. Utilizing adsorption to recover Li from aqueous solutions is an economical and eco-friendly approach. Nonetheless, most adsorbents lack specificity and exhibit low selectivity for individual metals. Therefore, it is crucial to explore novel adsorbents for selective separation of lithium from aqueous solutions [31].

Ion-imprinted polymers (IIPs) are adsorption materials that selectively target specific ions [32,33]. Using ion-imprinting technology, specific ions can be targeted by matching their charge number, radius, and spatial structure with those of the template ions [34,35]. Imprinted ionic polymers offer the advantage of exhibiting high selectivity for the target ion, which can be significantly greater, by several orders of magnitude, compared to nonimprinted polymers [33]. Additionally, they can be synthesized with specific properties such as high mechanical strength, thermal stability, and chemical resistance [33]. The ion-imprinted polymer technique has gained significant attention in recent years owing to its potential for the efficient extraction and recovery of metals from aqueous solutions. However, despite its potential, there is a scarcity of research regarding the implementation of this technique for the selective extraction and preconcentration of lithium ions [36,37,38,39]. In imprinted polymers, the adsorption process, specifically regarding lithium ions, is significantly impacted by the choice of the functional monomer. The chemical structure of the functional monomer played a crucial role in promoting the coordination of Li ions, thereby influencing the overall efficiency and effectiveness of the adsorption process. Therefore, it is essential to carefully examine and evaluate the functional monomers to optimize lithium-ion adsorption, as there are no universal rules for this process. Functional monomers typically achieve selective analyte retention on the polymer through hydrogen bonding or ionic interactions depending on the solvent and pH of the processed sample. Response surface methodology (RSM) is frequently used for the parameter assessment and investigation of interactive effects [40,41].

Budnicka et al. conducted a comprehensive literature review of recent publications on IIPs that target alkali and alkaline earth metal ions. Their review provided valuable insights into the complex formation of these polymers with a range of organic ligands, including macromolecular and supramolecular materials. In particular, supramolecular entities such as crown ethers (e.g., 12-Crown-4 and Benzo-12-Crown-4) and calixarenes (e.g., [4] arene) have been shown to enhance the selectivity of IIPs for lithium ions [42]. Several research studies have shown that the utilization of crown ethers as macrocyclic hosts can enhance the stability and selectivity of metal ions when compared to their open-chain counterparts [43,44]. Crown ethers, which belong to a specific category of ionic complexants, are cyclic chemical compounds featuring multiple ether groups that can selectively bind to cations. These compounds are widely employed as functional monomers for the development of IIPs specifically designed for lithium adsorption [45,46]. Warnock et al. developed a tunable polynorbornene network with host–guest interactions by copolymerizing 12-crown-4 ligands for ion selectivity, poly(ethylene oxide) side chains to control the water content, and a crosslinker to form robust solids at room temperature. Single salt transport measurements revealed an unprecedented reverse permeability selectivity (~2.3) for LiCl over NaCl [47]. The extraction of lithium from brine sources involves selecting a suitable size of crown ether (CE) for the target cation. In aqueous resources, the concentrations of Mg^2+^ and Na^+^ are typically elevated, posing a significant challenge for achieving selectivity over Li^+^. Based on multiple research investigations, it has been found that ^14^C_4_ is currently the most well-matched cavity size for Li^+^ in terms of diameter. Consequently, it induced the least conformational change in the CE, making it the preferred choice [48,49,50]. However, the relative affinities of complexes with both Na^+^ and Li^+^ ions depend on the chemical environment [35,36,37,38], as exemplified by the 12-crown-4 ether (^12^C_4_) ligand [47]. Incorporating crown ethers into supported liquid membranes has been reported by certain researchers to achieve the selective transport of Li^+^ ions [51,52]. Huang et al. described novel photo-responsive lithium-ion imprinted polymers (P-IIPs) based on the surface of mesoporous carbon nitride C_3_N_4_, by using a mixture of crown ethers and azobenzene derivatives as functional monomers [53]. In the presence of Na^+^, K^+^, and Mg^2+^, P-IIPs exhibit ideal selectivity and adsorption properties for Li^+^. In addition, saturated P-IIPs with Li^+^ can be regenerated with UV light [53]. Nevertheless, it should be noted that crown ethers, despite their utility, are not chemically bound to the membrane and may potentially leach into the surrounding solution. The extraction of Li from salt lakes presents two primary challenges. Firstly, the utilization of expensive crown ethers as functional monomers incurs high costs, which poses a significant obstacle [48,49]. Secondly, the limited water solubility of these functional monomers hinders their ability to effectively coordinate with template ions, resulting in a reduced number of imprinted sites and diminished adsorption efficiency [50]. Considering the above-mentioned drawbacks in utilizing crown ethers for lithium extraction, it becomes crucial to explore the development of alternative ligands capable of selectively separating lithium from aqueous solutions. Consequently, novel functional monomers have been designed for the synthesis of lithium IIPs. In recent years, significant attention has been directed towards phthalocyanines as macrocyclic host molecules [54]. Metal ions can synchronize with the nitrogen atoms in the concavities of the core. To maintain stable binding and selectivity, the metal phthalocyanine core should suitably match the target ions [55]. The polymerization of ethylene glycol dimethacrylate (EGDMA) with an optional copolymer and a lithium chelate monomer lithium produces lithium-imprinted polymers [52]. Phthalocyanines serve as highly effective compounds with macrocyclic structures, functioning as hosts that exhibit remarkable stability in binding and selectivity towards Li ions.

In this study, a novel Li^+^-ion-imprinted polymer was developed, utilizing 2,9,16,23-tetra-(4-methacrloyloxyphenoxy) phthalocyanine as a lithium chelate monomer, with the primary objective of selectively separating Li^+^ from the waste generated by desalination plants using reverse osmosis techniques. The synthesis and characterization of phthalocyanine acrylate are for the subsequent polymerization process, to create Li-imprinted polymers. The metal uptake capacities were assessed through batch tests and by a solid-phase extraction (SPE) cartridge as a packed bed of the metal-imprinted polymers to evaluate their performance under dynamic conditions.

## 2. Materials and Methods

### 2.1. Reagents

All chemicals and solvents employed in the proposed research were analytical reagents, and ultrapure water was used throughout the experiments. The solvents used in this study were provided by reliable suppliers. 4-Nitrophthalonitrile was prepared and purified according to the methods described in our previous research [56,57]. N,N-dimethylaminoethanol (DMAE), 1,8-diazabicyclo[5.4.0]undec-7-ene (DBU), Hydroquinone, methacryloyl chloride, ethylene glycol dimethacrylate (EGDMA, 98%), and α-α′-Azoisobutyronitrile (AIBN, 98.5%) were purchased from Sigma-Aldrich (Steinheim, Germany). Lithium chloride (LiCl) (98%) was purchased from Techno pharmachem (Delhi, India). Nitric acid (HNO_3_), ethyl ether (C_4_H_10_O, 95%), and anhydrous sodium sulfate (Na₂SO_4_, 99% purity) were supplied by Merck KGaA (Darmstadt, Germany). The solvents used in this study were provided by reliable suppliers. HPLC-grade acetonitrile (ACN) and methanol were supplied by Fisher Scientific Co. (Hampton, NH, USA). The Millipore purification system at the Industrial Waste Treatment Lab (Department of Environment, KAU) provided ultrapure water. The system and MPK01 filter were manufactured by Millipore (Fontenay-sous-Bois, France).

### 2.2. Instruments

The Fourier transform infrared (FT-IR) spectra of the polymer particles were investigated using IRAffinty-1 Spectroscopy SHIMADZU (Kyoto, Kansai, Japan) in the range of the 4000–400 cm^−1^ region. For scanning electron microscopy (SEM), a Quanta 250 (Waltham, MA, USA) was used to examine the morphology of the polymer particles. Surface area analysis and average pore diameter measurement of the IIPs were conducted using an Autosorb-1-C chemisorption-physisorption analyzer (Quantachrome, Boynton Beach, FL, USA). To wash Li from the IIPs during the elution step, MAX Empty SPE Cartridges with two frits from JVLAB (Mainland, China) were used. ^1^H NMR and ^13^C NMR spectra were recorded on Agilent VNMRS 500 MHz with TMS as the internal standard. Mass spectra were measured on a Bruker Microflex MALDI-TOF/MS mass spectrometer. Inductively coupled plasma optical emission spectroscopy (ICP-OES) with a vertically orientated torch from the Agilent 5110 VDV series (Santa Clara, CA, USA) was used for the determination of Li and other elements in a brine sample. A digital ultrasonic cleaner (JPS-24AD, 3 L, Moscow, Russia) was used to disperse the mixtures and remove oxygen from the solution. The polymerization reactions were conducted in an oil bath. Grinding of IIP particles to a minimum granularity of 0.1 um was achieved using a 0.4 L Laboratory Pulveriser Ball Mill Small Planetary Ball Grinding Mill Machine (DECO, Hunan Yueyang, China) with grinding jars compatible with PTFE (Teflon) and ZrO_2_ (zirconium oxide) balls. Particle sizes ≤ 38 µm were obtained using a 400 mesh Stainless Steel Screen Cell Strainer (55 × 28 mm) (Hunan Yueyang, China).

### 2.3. Synthesis of 4-(4-Hydroxyphenoxy)benzene-1,2-dicarbonitrile

**Figure 1:**
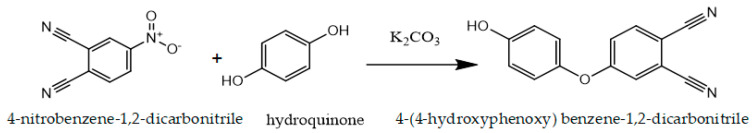
Synthesis of 4-(4-hydroxyphenoxy) benzene-1,2-dicarbonitrile.

4-(4-hydroxyphenoxy)benzene-1,2-dicarbonitrile was synthesized according to the reaction scheme shown in Figure 1.

A mixture of hydroquinone (4 g, 36.1 mmol), potassium carbonate (15 g, 108.3 mmol), and dry DMSO (60 mL) was stirred at room temperature for 20 min. Subsequently, 4-Nitrophthalonitrile (2.5 g, 14.5 mmol) was added to the reaction mixture, which was submitted to continuous stirring at 100 °C for 8 h. After cooling the mixture to room temperature, it was poured into 1 M HCl solution (300 mL). The resulting precipitate was filtered, washed with water, and dissolved in diethyl ether. The resulting solution was washed with water until the aqueous phase became neutral. Following the extraction process, the organic phase was dried using sodium sulfate. Diethyl ether was evaporated, and the resulting solid was recrystallized with methanol.

Yield: 2.7 g (79%); ^1^H NMR (500 MHz, DMSO-*d*6, 25 °C), δ 8.58 (br s, H), 8.01 (d, H), 7.54 (d, H), 7.37 (dd, H), 7.06 (m, ^2^H), 6.98 (m, ^2^H) ppm. ^13^C NMR (126 MHz, acetone-*d*6, 25 °C), δ 162.74, 146.27, 135.86, 121.93, 121.30, 121.06, 117.22, 116.80, 115.58, 115.14, 108.07 ppm. FT-IR υ_max_ (cm^−1^): 3380 (O-H), 3080 (Ar-CH), 2238 (C≡N), 1245 (O-C).

### 2.4. Synthesis of Phthalocyanine Derivatives

#### 2.4.1. Synthesis of 2,9,16,23-Tetra-(4-hydroxyphenoxy) phthalocyanine

**Figure 2:**
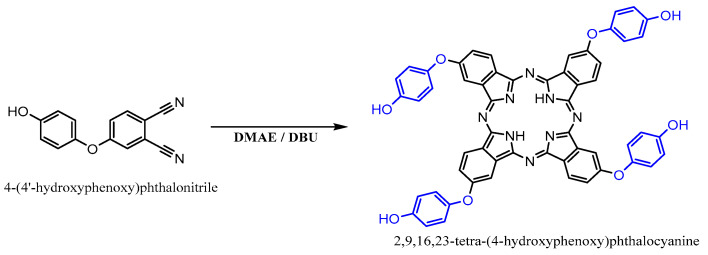
Synthesis of 2,9,16,23-tetra-(4′-hydroxyphenoxy) phthalocyanine.

2,9,16,23-tetra-(4′-hdroxyphenoxy) phthalocyanine (THPc) was synthesized according to the reaction scheme shown in Figure 2.

4-nitrobenzene-1,2-dicarbonitrile (2 g, 8.47 mmol) was dissolved in dry DMAE (30 mL). The temperature was then increased to 90 °C, and ten drops of DBU were added to the reaction mixture. Subsequently, the temperature was increased to 150 °C and the mixture was stirred for 24 h under N_2_. After cooling, the solutions were dropped into ethyl alcohol (40 mL) and the resulting precipitate was filtered off. The crude product was successively treated with diethyl ether, dichloromethane, and hexane before being dried under reduced pressure. Further purification of the crude product was achieved via column chromatography on silica gel with acetone, resulting in the formation of a green solid.

Yield: 1.26 g (63%); ^1^H NMR (500 MHz, acetone-*d*6, 25 ºC), δ 8.59 (s, ^4^H), 7.13- 6.43 (m, ^28^H), −7.70 (s, ^2^H) ppm. FT-IR υ_max_ (cm^−1^): 3293 (N-H), 3200 (O-H), 3031 (Ar-CH). UV-Vis (DMF): λ_max_/nm: 330, 612, 674. MALDI-TOF, (*m/z*) calcd.: 946.25; found: 947.27 [M+H]^+^.

#### 2.4.2. Synthesis of 2,9,16,23-Tetra-(4-methacrloxyphenoxy) phthalocyanine

**Figure 3:**
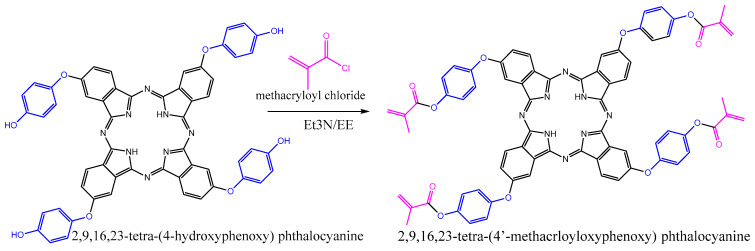
Synthesis of 2,9,16,23-tetra-(4-methacrloyloxyphenoxy) phthalocyanine.

2,9,16,23-tetra-(4-methacryloxyphenoxy) phthalocyanine (TMAPc) was synthesized as shown in Figure 3, following the procedure reported by Longo et al. [58].

A mixture of TMAPc (1 g, 1.05 mmol), triethylamine (Et_3_N) (0.81 g, 8.0 mmol), and methacryloyl chloride (0.84 g, 8.0 mmol) in 20 mL of diethyl ether (EE) was stirred for 18 h at room temperature under a nitrogen atmosphere. The reaction mixture was then added to approximately 20 mL of water and the resulting solution was extracted with CH_2_Cl_2_. The organic solution was dried using anhydrous sodium sulfate. After evaporating the solvent under reduced pressure, the crude product was obtained and further purified by washing with n-hexane.

Yield: 0.91 g (72%). ^1^H NMR (400 MHz; CDCl_3_; Me_4_Si): δH, ppm 7.29-7.04 (m, ^28^H), 6.49 (m, ^4^H), 5.89, (m, ^4^H), 2.21-1.88 (m, ^12^H). FT-IR (ATR system): ν, cm^−1^ 2957, 1735, 1616, 1499, 1474, 1320, 1187, 1127, 1012. UV-vis (CHCl_3_): λ_max_, nm 285, 342, 606, 638, 665, 700. MS (ESI): *m/z* 1219 (calcd. for [M +H] ^+^ 1219).

### 2.5. Lithium-Ion-Imprinted Polymer Preparation (Li-IIP)

**Figure 4:**
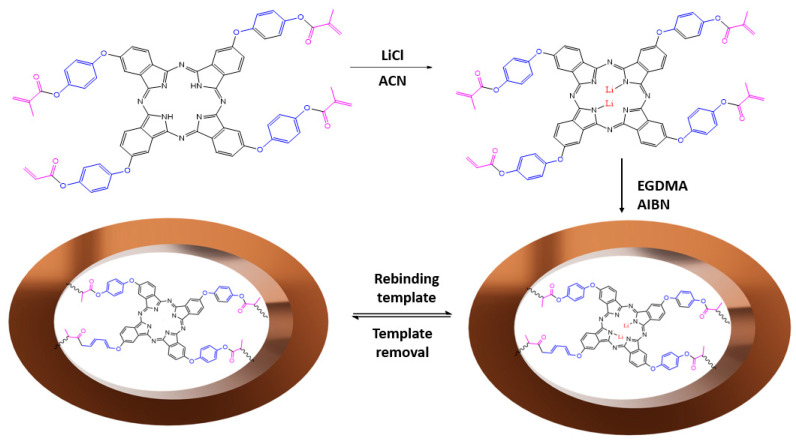
Schematic illustration of Li-IIP synthesis steps and Li removal from IIP binding sites.

The Li-ion-imprinted polymer (Li-IIP) was synthesized using the precipitation polymerization technique, following the methodology developed by Qronfla et al. [59] with some modifications. In a 10 mL glass test tube, 2 mmol of LiCl (template) was mixed with 5 mL of acetonitrile and 4 mmol of TMAPc (functional monomer). The test tubes were then placed in an ultrasonic bath at room temperature for 30 min. Subsequently, the test tube was promptly sealed, and the solution was purged with nitrogen gas to remove any dissolved oxygen. Subsequently, 20 mmol EGDMA (crosslinker) and 478 µL AIBN were injected into the solution. Sonification of the solution was continued for an additional twenty minutes. Following the deoxygenation process, the reaction mixture was heated in an oil bath at 60 °C for 24 h and continuously purged with N_2_ gas. A flowchart of the IIP creation procedure is shown in Figure 4.

After 24 h, the polymer was filtered to remove solvent. The resulting IIP powder was washed with methanol to eliminate any unreacted materials and then leached with 1 mol/L HNO_3_ until the washed solution was free of lithium ions. Finally, the lithium IIP was washed with distilled water until it reached a neutral pH. The final IIP was then dried in an oven at 60 °C for 24 h. Subsequently, the polymer was crushed and sieved at room temperature, yielding particles of 38 µm or smaller. The non-imprinted polymer (NIP) was synthesized similarly but without the presence of the template molecule. The IIP was dried at 60 °C for 6 h and stored at room temperature. Table 1 presents the procedures used to generate the IIP and NIP for Li.

**Figure 5:**
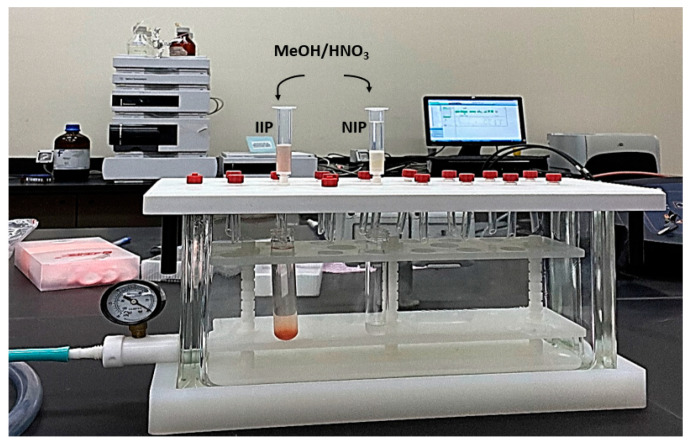
IIP or NIP wash process.

In the experimental procedure, a 3 mL SPE cartridge was employed for the elution process. The cartridge was packed with the optimized sorbent mass of the imprinted polymer (IIP) or non-imprinted polymer (NIP), positioned between two polyethylene frits. The compacted powder specimen was extracted through the stationary phase under optimal elution conditions using an SPE vacuum manifold at controlled extraction speed and sample flow, as illustrated in Figure 5. To remove lithium ions, IIPs were leached with 0.1 M HNO_3_ four times and twice with distilled water. HNO_3_ is preferred because it gives fewer matrix effects than HCl for the subsequent analysis by ICP-OES. The eluate was then quantified using inductively coupled plasma optical emission spectrometry (ICP-OES), following the method described by Tao et al. [60]. The procedure was repeated four times, covering the conditioning phase during which Li became undetectable.

### 2.6. Polymer Characterization

The structures of the IIP and NIP particles were characterized using FT-IR spectroscopy. Spectral analysis was performed within the wavenumber range of 500–4000 cm^−1^, with a spectral resolution of 2 cm^−1^. In addition, the morphologies of the polymer surfaces were examined by SEM. The dry polymer specimens were coated with a thin layer of gold before being subjected to SEM.

### 2.7. Binding Experiments

The lithium binding capacity of the polymer was assessed through batch adsorption tests conducted at various temperatures using a buffer solution of known composition. The procedure involved contacting 10 mg of the dried polymer with 10 mL of buffer solution under ultrasonication for 30–60 min at the desired temperature. To calculate the lithium uptake of the polymer, the metal concentration in the initial solution (C_0_) was compared with that in the solution after polymer treatment (Ce). The concentration of lithium ions in the solution was determined by inductively coupled plasma optical emission spectrometry (ICP-OES). The lithium uptake was calculated using the following equation [61]:

(1) $$Q_{e}=\frac{\left(C_{0}-C_{e}\right)\times V}{w}$$

where *Qe* (mg·g^−1^), *C*_0_ (mg·mL^−1^), and *Ce* (mg·mL^−1^) represent the adsorption amount, initial concentration, and equilibrium concentration of Li^+^, respectively. *V* (L) is the volume of the solution and *w* (g) is the weight of the polymer IIP or NIP used for the test. The tested Li-imprinted polymer was transferred to an SPE column to evaluate its metal binding characteristics under different conditions such as flow-through tests.

### 2.8. Metal Binding Selectivity

The separation factors of the Li-imprinted polymers were assessed by conducting batch tests and SPE column flow-through tests with synthetic brines containing known compositions of lithium and other metals. The uptakes of lithium and other metals were measured, and the selectivity factors (*α_Li/M_*) were calculated from their distribution coefficients (K_d_) using the following formula [62]:

(2) $$Selectivity\;separatiion\;factor\;\alpha_{Li/M}=\frac{Q_{Li}}{C_{Li}}\times \frac{C_{M}}{Q_{M}}$$

where *Q_Li_* and *Q_M_* represent the adsorption capacities of lithium (Li) and the other metals (M) in the polymer (meq/g polymer), respectively, and *C_Li_* and *C_M_* are the concentrations of Li and other metals in the brine (meq/L brine) being tested.

Li-imprinted polymers were subjected to batch experiments to assess their metal uptake capacity from synthetic brine containing Li^+^, Na^+^, K^+^, Ca^2+^, and Mg^2+^ ions. These tests were conducted under optimal pH conditions and temperatures. After evaluating the IIP performance using the synthetic ionic solution, the same IIP was employed to test real SWRO brine under optimal temperature and pH conditions.

### 2.9. Optimization of Li Extraction by IIP-SPE Using Experimental Design Approach

Optimization of Li extraction through IIP-SPE involved the selection of four key parameters: sample pH, sorbent mass (SM), elution volume (EV), and temperature (T) (the temperature maintained during adsorption or desorption). These parameters were selected based on Alshuiael’s prior work and the initial experimental tests [63]. The response surface statistical method was employed to design optimization experiments and control the process of adsorption and recycling of Li by MIP-SPE. The operational ranges of the selected variables are presented in Table 2. For this purpose, an orthogonal central composite design (CCD) with nine center points and a (24 + star) configuration was established. This design was achieved by combining the Statgraphics Centurion XVI software package version 16 and MATLAB R2019a version, ensuring rotatability of the design with α = 1.86792.

The proposed design (CCD) and backward algorithm for model building facilitated the performance of 18 experiments, each involving different combinations of the four factors. The responses focused on the adsorption and desorption of Li through the IIP-SPE. A second-order model was employed for the RSM, and the equation is as follows:

(3) $$\hat{Y}=\beta_{0}+\sum_{i=1}^{4}\beta_{ii}X_{i}^{2}+\sum_{i=1}^{3}\sum_{j=i+1}^{4}\beta_{ij}X_{i}X_{j}+\varepsilon$$

where $\hat{Y}$ denotes the predicted response of the process, and *β_0_*_,_ *β_i_*, *β_ii_*, and *β_ij_* are the regression coefficients of intercept, linear, quadratic, and interactive terms, respectively. *X_i_* and *X_j_* are levels of the coded levels of the factors (independent or control variables) and ε is the statistical error.

In the designed experiments, a 6 mL capacity SPE cartridge containing IIP in the range of 1–50 mg (factor x_2_ in Table 2) was placed between two frits. The III-SPE column was sequentially conditioned using an aqueous solution of 0.1 M NaOH which was passed through the column to exchange H^+^ with Na^+^ and the polymer was washed with a large excess of water to remove any free Na^+^ at a flow rate of 0.5 mL/min. Subsequently, a Li solution with a concentration of 5 mg/L was passed through the cartridge containing a mass of sorbent (x_2_) at a specified pH (factor x_1_) and T temperature at which the polymer is pretreated before filling the column (factor x_3_), with a variable elution volume between 5 and 10 mL (factor x_4_). The resulting eluant was analyzed by ICP-OES. The adsorption efficiency of the IIP was determined using the following equation, where *C_i_* and *C_f_* represent the concentrations of Li in the solution before and after adsorption, respectively [64]:

(4) $$Adsorption\;efficiency\left(\%\right)=\frac{\left(C_{i}-C_{f}\right)}{C_{i}}\times 100$$

To enhance the extraction of Li from IIP-SPE and reduce the number of tests required, the quantities of Li loaded onto the column during the adsorption phase were subjected to an elution phase, considering the operational desorption factors listed in Table 2. The experiments were organized into 18 series using the software tools Statgraphics version 16 and MATLAB R2019a version. The percentage of extracted Li was calculated using the following equation [65]:

(5) $$Extraction\;efficiency\left(\%\right)=\frac{C_{ext}}{C_{ads}}\times 100$$

where *C_ext_* and *C_ads_* are the Li concentration extracted and adsorbed, respectively.

### 2.10. Recycling Performance

To evaluate whether the material can be further applied in industry, the reusability of the ion imprinted polymer was tested under conditions of high selectivity for Li^+^ and high adsorption efficiency at the optimal temperature and pH. ICP-OES analysis was performed to determine the extraction and desorption efficiencies of Li^+^. The polymers were vacuum-dried overnight at 60 °C and subsequently reused for Li^+^ adsorption. This sorption–desorption cycle was repeated until Li^+^ was no longer detectable.

## 3. Results and Discussion

### 3.1. Characterization of Molecular Imprinted Polymer

#### 3.1.1. Scanning Electron Microscope (SEM)

**Figure 6:**
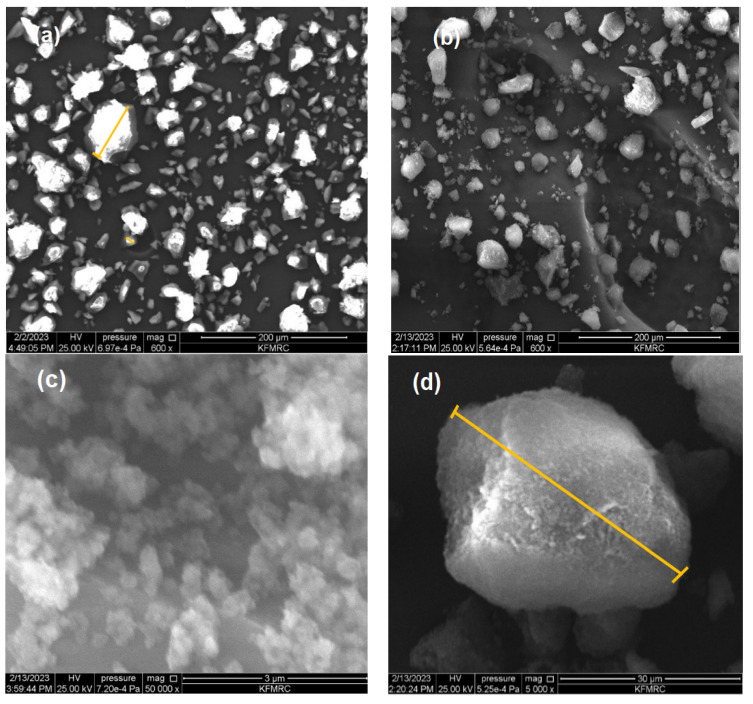
SEM Images of unleached IIP (**a**) and leached IIP at different magnifications (**b**–**d**).

The characteristics of IIPs, like other metal-ion sorbents, depend significantly on their morphology, including their shape and porous structure. Figure 6a–d display the SEM images of the Li-imprinted polymer before washing and after washing, revealing its morphology at various magnifications. The SEM images reveal a noticeable difference in morphology between the leached and unleached IIP (200 μm in × 200, 30 μm in × 500, and 3 μm in 50,000 magnification). The adsorption of lithium on IIP led to a visible change in the surface structure, as depicted in Figure 6a,b. SEM analysis of the polymer revealed an irregular shape, yet it exhibited a very regular and consistent porous surface structure for the IIP, appearing rough and mound-like. The surface of the IIP exhibited evenly dispersed local pores. Comparing the leached IIP (Figure 6b–d) to the unleached IIP (Figure 6a), it exhibited a rough and porous surface, suggesting the presence of empty binding sites for the target ions. This increased surface area enhanced the adsorption capacity and facilitated the capture of target ions. The removal of template ions results in increased roughness of the imprinted polymer surface [66]. The spherical shape of the polymer particles can be attributed to the precipitation polymerization mechanism [67]. Moreover, the porous texture contributed to improved adsorption by increasing the superficial area and exposing the surface binding sites. The adsorption of lithium as a target ion during polymerization is likely the cause of the morphological variations between leached and unleached IIPs.

The SEM images of the unleached IIP (Figure 6a) clearly show an even distribution of the target ion, which is strongly bonded to the surface. The presence of bound lithium ions results in a rough and aggregated structure on the surface of unleached IIPs [68]. In contrast, the leached IIPs obtained after cross-linking and imprinting processes exhibited an increased adsorption surface area with numerous micropores on the surface [68]. These micropores provide open areas that facilitate easy binding of the target ions, allowing for efficient adsorption on the leached IIPs.

#### 3.1.2. Surface Area and Porosity Analysis

**Figure 7:**
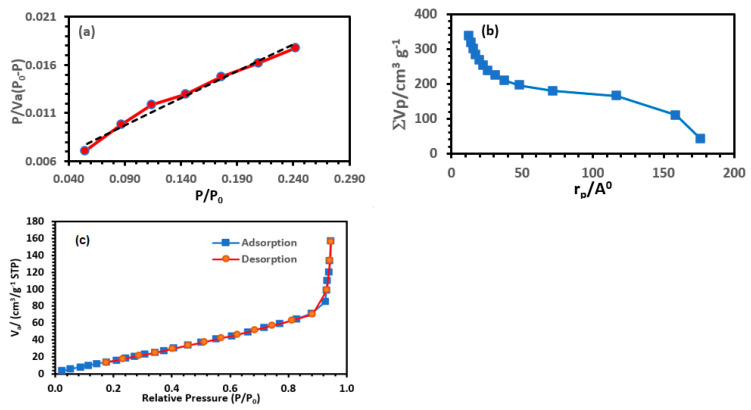
BET (**a**), BJH (**b**), and adsorption/desorption isotherms (**c**) plot of lithium-imprinted polymer.

Brunauer–Emmett–Teller (BET) theory was used to determine the specific surface area of the lithium-imprinted polymer, while the Barrett–Joyner–Halenda (BJH) theory was used to derive the pore volume and average pore diameter (Figure 7). The N_2_ adsorption capacity of lithium-ion-imprinted polymer (Li-IP) increased slowly at low pressures (P/P0 < 0.5) and sharply increased at high pressures (0.8 < P/P0 < 1.0). This indicates that the N_2_ isotherm of Li-IP is a type IV isotherm, which is characteristic of materials with abundant mesoporous structures. The surface area, BJH adsorption cumulative pore volume, and BJH adsorption average pore diameter of the prepared lithium-imprinted polymer were 106.03 m^2^/g, 0.387 cm^3^/g, and 3.817 nm, respectively.

#### 3.1.3. Fourier Transform Infrared Spectroscopy (FTIR)

The FTIR maps of the ion-imprinted polymer were swept at 4000~400 cm^−1^ using the KBr infrared spectroscopy method to study the functional groups of the adsorbent.

**Figure 8:**
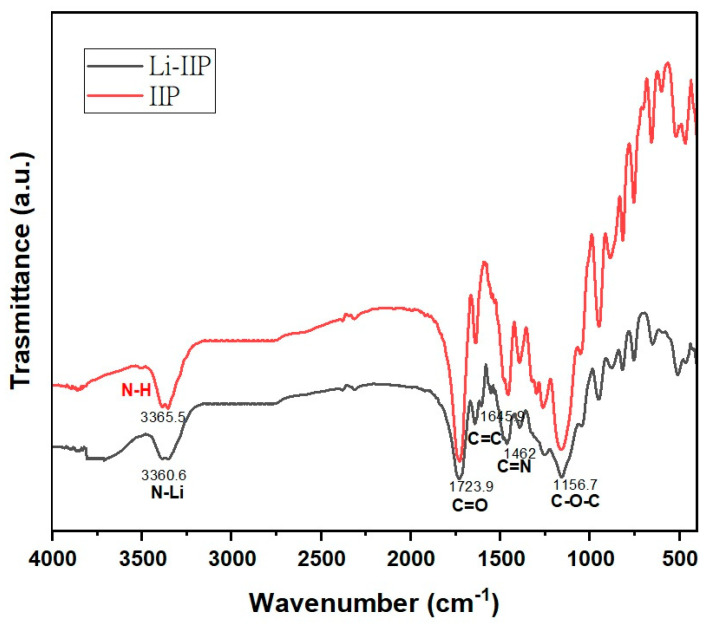
FT-IR spectra of the unleached (Li-IIP) and the leached IIP (IIP) ion-imprinted polymer.

A comparison of the IIP-Li and IIP spectra (Figure 8) revealed that the leaching process had no discernible impact on the functional groups of the imprinted ion polymer (IIP). This indicates a high degree of reproducibility of the IIP. Additionally, Işıkver et al. [67] conducted experiments comparing the spectra of the leached IIP with that of the control polymer, and intriguingly, the spectra displayed significant similarities. This finding suggests that the leaching process effectively removed ions without harming the polymer network. Specific vibrational peaks were observed in the FT-IR spectra of Li-IIP and IIP. The strong vibration at 1723 cm^−1^ corresponds to the ester carbonyl groups of EGDMA [69], and the absorption band at 1156 cm^−1^ corresponds to the C–O–C stretching vibration [70]. Furthermore, the peak at 1645 cm^−1^ indicated the successful grafting of the double bond onto the surface of the imprinted polymer. Upon meticulous analysis of the spectra (Figure 8), it was observed that the spectral features and functional groups of IIP exhibited minimal differences before and after adsorption. Nevertheless, there were some notable changes in the transmittance percentage of a few bands, as well as slight shifts in their exact positions, which were especially noticeable in the IIP after Li leaching. These changes suggest that the internal structure of the IIP undergoes modifications while capturing Li^+^ ions, resulting in shifts in the bands, particularly in the range of 3360–3365 cm^−1^. These shifts imply complexation of the NH functional groups of the phthalocyanine core with Li(I).

### 3.2. Adsorption Capacity

**Figure 9:**
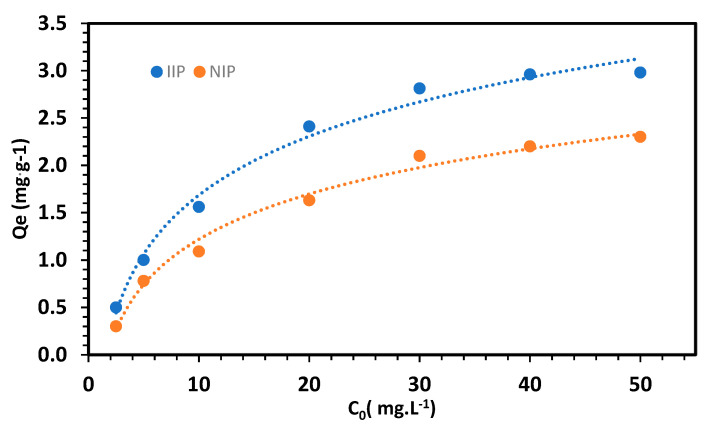
Adsorption capacity studies for the IIP and NIP.

As expected, IIP showed a higher adsorption capacity than NIP (Figure 9), which can be attributed to its specific imprinted sites on the polymer for the target analyte. This result led to the exclusive selection of IIP for the optimization of the extraction procedure. Subsequently, adsorption studies were performed to examine the adsorption behavior of both the IIP and NIP. The equilibrium adsorption data for Li on the imprinted polymers were analyzed using the Langmuir and Freundlich [71] isotherms, employing nonlinear Equations (3) and (4).

(6) $$\mathrm{Langmuir}:\;q=\frac{q_{m}\;k_{L}C_{e}}{1+k_{L}C_{e}}$$

where *q_e_*, *q_m_*, *k_l_*, and *C_e_* are the amount of Li adsorbed, maximum adsorption capacity, Langmuir constant, and concentration of Li at equilibrium, respectively.

(7) $$\mathrm{Freundlich}:\;q_{e}=K_{F}C_{e}^{\frac{1}{n}}$$

where *K_F_*, *C_e_*, and *n* are the measure of adsorption capacity, equilibrium concentration, and indicator of adsorption effectiveness, respectively.

The adsorption data for Li on the IIP showed a better fit to the Langmuir isotherm, with a correlation coefficient of 0.9765, outperforming the R^2^ value of 0.7675 obtained for the Freundlich isotherm. This outcome suggests that the adsorption of Li onto the IIP is uniform, indicating monolayer adsorption [72,73]. Based on the Langmuir isotherm, the maximum adsorption capacity was calculated to be 3.2 mg·g^−1^.

### 3.3. Characterization of SWRO Brine

Extensive physical and chemical analyses were conducted on brine obtained from a desalination plant using SWRO. The analysis covered several key parameters, including pH, salinity, total dissolved solids (TDSs), conductivity, and elemental composition. The results, along with data from other studies on SWRO brine, are compiled and summarized in Table 3.

In this study, the pH of the brine was approximately 8, indicating its alkaline nature. This observation is consistent with the pH values reported in other studies. In addition, analysis of the brine sample indicated noteworthy mineral concentrations, such as Na (32,575 mg/L), Mg (2863 mg/L), K (1680 mg/L), Ca (1675 mg/L), and Li (45.36 mg/L). Additionally, the study revealed relatively low concentrations of trace metals in the analyzed brine, including Ba (0.09 mg/L), Zn (0.625 mg/L), Fe (0.73 mg/L), Cu (1.234 mg/L), Pb (0.432 mg/L), and V (1.634 mg/L).

### 3.4. Optimization of PII-SPE Procedure by Experimental Design Approach

The results obtained for the adsorption and desorption of Li using the IIP-SPE procedure are presented in Table 4 for all CCD runs. The response values ranged from 65% to 97% for extraction and from 5.9% to 86.4% for retention.

The optimization results were analyzed using an analysis of variance (ANOVA), RSM, and desirability function to determine the significant factors, interaction effects, and optimal extraction conditions. Statistical estimators derived from the ANOVA were used to assess the adequacy of the reduced quadratic models (Table 5 and Table 6). The F-value, which measures the data variance, was employed to determine the statistical significance of the models. The reported F-values for the selected models in both the adsorption and desorption steps deviated significantly from unity, indicating reliable and high-level predictions based on the empirical data. The low *p*-values in both phases further confirmed the statistical validity of the models for predicting the response. The quality of fit for the polynomial model equation was expressed by the coefficient of determination (R^2^) and the adjusted coefficient of determination (R^2^adj) in ANOVA. R^2^ represents the proportion of variation in responses explained by the predictors in the model. Both the adsorption and desorption models demonstrated desirable R^2^ values of close to 1. The predicted R^2^ values aligned well with the adjusted coefficient of determination (R^2^adj), indicating an appropriate selection of factors influencing the efficiency of the IIP-SPE procedure.

The variability in adsorption was analyzed using ANOVA (Table 5), which individually examined each effect. The significance of each effect was determined by comparing the mean square error with an estimate of the experimental error. Three of the tested effects exhibited a *p*-value of less than 0.05, indicating a significant difference from zero at the 95.0% confidence level. The R-squared statistic revealed that the adjusted model accounted for 96.1623% of the adsorption variability. Comparing the models with different numbers of independent variables, the adjusted R-squared statistic was 78.2533%, which is considered more appropriate. The standard error of the estimate indicated a standard deviation of 12.6037 for residuals. A mean absolute error (MAE) of 4.44937 represents the average value of the residuals. The Durbin–Watson (DW) statistic was used to assess if there was any significant correlation in the residuals based on their order in the data file. A *p*-value greater than 5.0% suggests no indication of serial autocorrelation in the residuals at the 5.0% significance level.

For the desorption step, eight effects demonstrated a significant difference from zero at the 95.0% confidence level, as their *p*-values were less than 0.05. The R-squared statistic revealed that the adjusted model accounted for 97.3841% of the variability in the extraction. Comparing the models with different numbers of independent variables, the adjusted R-squared statistic was 85.1766%, which is considered more appropriate. The standard error of the estimate showed a standard deviation of 1.4799 for residuals. The mean absolute error (MAE) of 2.11177 represents the average value of the residuals. Additionally, with a *p*-value below 5.0%, there was a possibility of serial correlation at the 5.0% significance level.

In the following phase, the Multivariate Regression method was used to create empirical models for predicting Li using coded factors. The models were based on the significant regression coefficients (*p* < 0.05) obtained from the reduced quadratic models. The established models were then employed to estimate the Li adsorption and desorption efficiencies using the Li procedure. The experimental adsorption and desorption data were fitted to second-order models and are expressed as follows:

Adsorption step:

Adsorption efficiency (%) = −223.858 + 39.1303 * x_1_ + 4.45311 * x_2_ + 3.16201 * x_3_ − 2.30639 * x_4_ − 2.97438 * x_1_^2^ + 0.200319 * x_1_ * x_2_ + 0.0545345 * x_1_ *x_3_ + 0.105683 * x_1_ * x4 − 0.0539011 * x_2_^2^ − 0.0294655 * x_2_ *x_3_ − 0.0156051 * x_2_x_4_ − 0.0292931 * x_3_^2^ + 0.0132874 * x_3_ * x_4_ − 0.00722896 * x_4_^2^ (8)

Desorption step:

Extraction efficiency (%) = −48.5515 + 8.84371 * x_1_ + 0.802575 * x_2_ + 1.28435 * x_3_ + 13.3157 * x_4_ − 0.942694 * x_1_^2^ + 0.0480596 * x_1_ * x_2_ + 0.0615298 * x_1_ * x_3_ + 0.130042 * x_1_ * x_4_ − 0.0214192 * x_1_^2^ − 0.0101109 * x_2_ * x_3_ + 0.0415523 * x_2_ * x_4_ − 0.0129525 * x_3_^2^ + 0.0107762 * x_3_ * x_4_ − 0.814868 * x_4_^2^\ (9)

where x_1_, x_2_, x_3_, and x_4_ are the values of four independent variables (pH, sorbent mass, temperature, elution volume).

**Figure 10:**
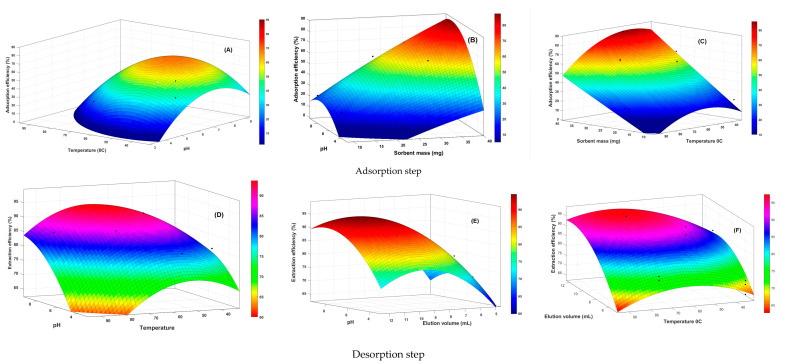
RSM showing the interaction between the independent factors (**A**,**D**) pH and temperature, (**B**) pH and sorbent mass, (**C**) temperature and sorbent mass, (**D**) pH and elution volume, (**E**) elution volume and temperature, and (**F**) elution flow rate and sorbent mass.

The response surface models were visually represented through RSM plots, which showed the interaction effects of the operational variables on the response factors. Figure 10 shows 3D response surface plots illustrating these relationships.

Optimization of the pH value in SPE or batch-test procedures is essential to enhance intermolecular interactions such as ionic interactions, hydrogen bonding, and hydrophobic interactions between the analytes and the ionic imprinted polymer (IIP). Moreover, the pH value significantly influences the form of the metal ions and the charge distribution on the surface of the adsorbing material, consequently exerting a substantial impact on the adsorption process. In addition, its interactive relationship with the other three independent variables also had an important effect on the adsorption and desorption efficiencies, as shown in the Figure 10A–F. Figure 10A,B illustrate that the adsorption of Li^+^ by the polymer (IIP) increases when the pH is within the range of 7 to 8. However, a sharp decline in the Li^+^ adsorption efficiency of IIP was observed when the pH dropped below 7. In solutions with a pH below 6, many H^+^ ions compete with Li^+^ which prevents it from establishing a coordination bond with the functional groups of the polymer, particularly the amine functions of the four iso-indole units of phthalocyanine. Consequently, the tendency for metal ion–Phtlocyanine complex formation increases as pH rises. These findings are consistent with the research conducted by Behbahani et al. [77] on nano-IIPs, where they also investigated various pH values. They observed a similar trend of increased adsorption as the pH increased from 2 to 8. Furthermore, they noted that reducing the pH of the solution resulted in a decrease in the quantitative retention of the sorbent, which was attributed to electrostatic repulsion between the protonated active sites of the sorbent and positively charged metal species. Upon reaching a pH level greater than 8, the adsorption capacity of the IIP for Li^+^ started to decline. This decrease in the adsorption capacity can be attributed to the increased quantity of OH^−^ ions in the alkaline solution. The presence of higher OH^−^ concentrations likely led to the hydrolysis and precipitation of Li^+^, resulting in a reduced concentration of free Li^+^ and, consequently, a lower adsorption capacity. Therefore, a pH value of 6.0 in aqueous solution was selected as the optimal pH for the subsequent experiments.

The synergistic influence of the sorbent mass and various other factors (pH, temperature, and elution volume) demonstrated that the optimal adsorption of Li^+^ occurred within a pH range of 7 to 8 (as depicted in Figure 10B,F) and at temperatures ranging from 40 °C to 60 °C (Figure 10C). It was observed that adsorption improved as the sorbent mass increased within the range of 30–40 mg (Figure 10C). This indicates that a higher mass of the polymer provided an increased number of binding sites for the template, resulting in the quantitative retention of Li^+^. Therefore, increased sorbent mass enhances the adsorption capacity and improves the efficiency of Li^+^ adsorption. Figure 10A,C demonstrate the impact of temperature on the adsorption capacity of IIP for Li^+^. Generally, an increase in temperature leads to a corresponding increase in adsorption capacity. This phenomenon is consistent with the findings of Al-Ajj et al. [78], who suggested that the increase in adsorption capacity could be attributed to the enhanced diffusion rate of the adsorbate molecules owing to the reduction in solution viscosity at higher temperatures. Moreover, it is plausible that elevated temperatures enhance the flexibility of the phthalocyanine core in the imprinted polymer, creating more accessible binding sites for ions to interact with. However, the relationship between temperature and adsorption capacity showed an opposite trend in the temperature range above 50 °C, as shown in Figure 10A,C. The decline in adsorption efficiency at temperatures above 50 °C can be ascribed to multiple factors. First, the temperature weakens the bonds between the metal ions and the polymer surface, leading to a reduction in the adsorption capacity. Second, temperature affects the solubility of metal ions in the solution, potentially impeding their adsorption onto the polymer surface. Finally, higher temperatures increase the mobility of the adsorbed metal ions, leading to diminished surface coverage and overall adsorption capacity.

During the desorption phase, the elution volume plays a pivotal role in achieving effective extraction. Specifically, an increased elution volume (as depicted in Figure 10E,F) disrupts the interaction between the analyte and sorbent, making it easier for the analyte molecules to be washed away. Consequently, the affinity for the sorbent surface diminishes. These findings are consistent with those of Sangai et al. [79].

**Figure 11:**
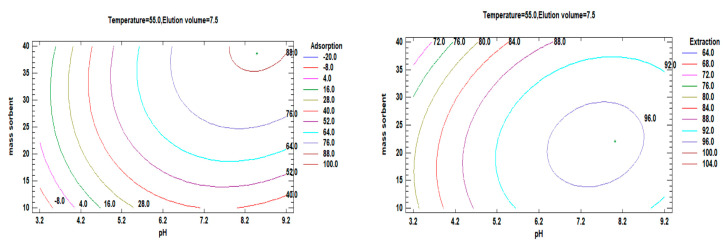
Contours of estimated response surface and showing the region that maximizes adsorption and desorption for a fixed temperature and elution volume.

Figure 11 illustrates the optimization process to determine the combination of factor levels that maximizes adsorption efficiency and extraction within specific regions. This can be achieved using either a single value or multiple factors. The estimated response surface contours depict the regions where adsorption and desorption are maximized at a fixed temperature and elution volume. The results from these contours indicate that high adsorption of Li^+^ occurs at an estimated pH value of 8.49 with a sorbent mass of 38.67 mg. Similarly, the best Li^+^ extraction is obtained at an estimated pH value of 8 with a sorbent mass of 22 mg, following a similar trend.

**Figure 12:**
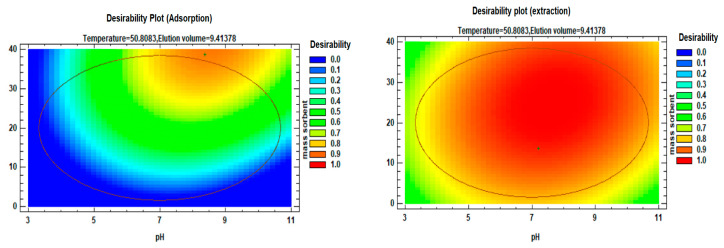
Desirability profiles and predicted values for factors affecting the adsorption and extraction of Li(I).

The desirability profile and predicted values (Figure 12) were employed to identify the optimal conditions for each factor based on their desirability. A desirability value of 1 was set as the target to provide guidance for estimating the conditions required to achieve the highest possible signal enhancement.

Following the acquisition of regression models, analysis of variance (ANOVA) was conducted to evaluate the response surface quadratic model, and the optimum values for the selected parameters were determined for both the adsorption and desorption processes. For the adsorption process, the optimum operating conditions were as follows: sample pH of 8.49, sorbent mass of 38.67 mg, temperature of 45.5 °C, and 5.0 mL of the elution sample. Similarly, for the desorption process, the optimum operating conditions were as follows: a sample pH of 8, sorbent mass of 22 mg, temperature of 64 °C, and 9.7 mL of elution solvent. These conditions were identified through a systematic approach based on the obtained regression models, ANOVA analysis, and consideration of the desirability plot to achieve the desired efficiency in both the adsorption and desorption processes.

### 3.5. Selectivity of Li-Imprinted Polymers

Two synthetic brine samples were used to evaluate the selectivity of the prepared Li-printed polymers. One sample had low concentrations of Na^+^ and K^+^, whereas the other contained high concentrations of Na^+^ and K^+^. The optimal conditions, determined by considering the pH, mass of the sorbent, temperature, and volume of elution, were used for the selectivity tests. Additionally, a Li-imprinted polymer derived from a lithium chelate phthalocyanine monomer was evaluated using flow-through SPE under the same conditions. In batch tests, the capability of the Li-imprinted polymer to adsorb lithium in the presence of similar concentrations of Ca^2+^ and Mg^2+^ was evaluated using synthetic brine containing low concentrations of K^+^ and Na^+^.

Li adsorption tests on the prepared polymers were performed in both batch mode and continuous flow using SPE columns. The Li-binding capacity of the polymer in the SPE column was like that obtained from the batch tests, with values ranging from 2.8 to 3.2 mg Li^+^ per gram of polymer. The Li capacity results obtained from solutions with a concentration of 50 ppm of Li in a buffer solution (NH_4_Cl/NH_4_OH) at pH 8 percolated on the SPE columns at a temperature of 45 °C and a flow rate of 0.5 mL/min for 20 min showed slightly lower values (~2.8 mg Li^+^/g polymer) compared to the previous data (~3.2 mg Li^+^/g polymer). This observation can be explained by the interference of the high concentration of NH_4_^+^ in the buffer, which affects the binding of Li^+^. The selectivity factor (α_Li/M_) was determined for synthetic brines (I) containing a mixed ternary solution of 50 mg/L Li^+^, 50 mg/L Na^+^, and 50 mg/L K^+^ in a flow-through SPE column. The cross-selectivity evaluation results for the IIP packed in the SPE column are listed in Table 7. To simulate the composition of the residues of SWRO, we determined the selectivity factors of Li^+^ in synthetic brines (II) with high concentrations of Na^+^ and K^+^ in batch mode. The brine composition tested contained 50 ppm Li^+^, 30,000 mg/L Na^+^, and 1500 mg/L K^+^.

As shown in Table 7, the separation factors for Li^+^ versus Na^+^ and Li^+^ versus K^+^ in brine (I) were determined to be 3.6 and 3.4, respectively. However, the Li vs. Na selectivity decreased from 3.6 to 2.6, and the Li vs. K selectivity in brine (II) decreased from 3.6 to 2.3. These results highlight the preference of the polymer for adsorbing Li^+^ over Na^+^ and K^+^, showing its selective binding to Li over Na and K. To evaluate the selectivity of a representative Li-imprinted polymer in the presence of Ca^2+^ and Mg^2+^, batch experiments were performed on synthetic brine (III) containing 50 mg/L Li^+^, 2500 mg/L Mg^2+^, and 1500 mg/L Ca^2+^. The selectivity factors of the Li-imprinted polymer determined in brine (III) at 45 °C and pH 8 are listed in Table 8.

According to Table 7, the data reveal that the polymer has a higher affinity for Mg^2+^ than for Li^+^, as indicated by a selectivity separation factor slightly lower than 1. This preference can be attributed to the ability of the polymer to form complexes with Mg, specifically with phthalocyanine monomers incorporated into its structure. Consequently, the presence of Mg^2+^ in brine impedes the adsorption of lithium by the polymer. Nevertheless, the polymer exhibited a selectivity separation factor greater than 1 for Li^+^ over calcium (Ca^2+^). Therefore, to ensure an efficient lithium extraction process, it is crucial to perform preliminary separation of Mg^2+^ from the brine before the extraction of lithium.

### 3.6. Reusability of IIP Packed in the SPE Column

**Figure 13:**
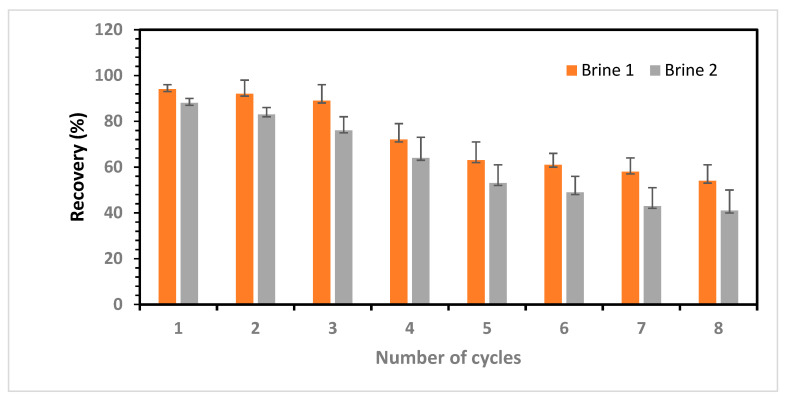
Adsorption stability and regeneration performances of IIP after multiple adsorption–desorption cycles.

To evaluate the reusability of the IIP cartridge, brine samples (brine (I) and brine (II)) previously used in selectivity experiments were subjected to eight adsorption and desorption cycles with the same IIP. Following each cycle, the polymer was regenerated using 0.25 M HNO_3_, and samples of the acidic eluent were subjected to lithium content analysis using ICP-OES. Figure 13 illustrates the stability and regeneration performance of the IIPs after several adsorption–desorption cycles.

The findings indicate that the IIP demonstrates consistent efficiency during the first three adsorption–desorption cycles, maintaining 89% of the initial efficiency for brine (I). However, in Cycle 3, the efficiency of the IIP decreased to 76% of the initial efficiency of brine (II) (Figure 13). The reduced efficiency in Cycle 3 (brine II) could be attributed to the heightened competition for binding sites, exacerbated by the extremely high salinity of the brine. Additionally, the presence of intermolecular forces that cause electrostatic bonding among the ions in the brine further hinders the adsorption of single Li^+^ ions to the binding sites. These results indicate that IIPs are promising candidates for Li^+^ separation and concentration from brine with relatively high Na^+^/Li^+^ and K^+^/Li^+^ ratios. These findings are consistent with the previously determined Li selectivity factors for imprinted polymers in the two brine compositions.

### 3.7. Batch Studies of SWRO Brine

Once the IIP was characterized and its performance was evaluated using a synthetic ionic solution, the subsequent phase was applied to a real SWRO brine at a temperature of 45 °C and a pH of 8. To ensure the reliability, performance, and selectivity of the IIP, the brine sample was spiked with three low-concentration levels of lithium.

As indicated in Table 9, the recovery of Li from spiked samples ranged from 62.8% to 71.53%. The % removal of Li in the brine was 11.6% and 21% lower than the % removal of Li^+^ in the synthetic brine (II) solution. This decrease in the adsorbed lithium recovery can be attributed to the ionic complexity of the brine and the presence of electrostatic bonds between the ions, which hinder the selectivity of the IIP for the specific ion.

## 4. Conclusions

In summary, the main focus of this study is to address a notable environmental issue related to SWRO brine. A novel technique that combines an ion-imprinted polymer (IIP) and SPE is proposed for the selective adsorption of lithium ions from reverse osmosis brine. IIPs were synthesized using a bulk polymerization protocol and a non-covalent approach, utilizing lithium as the template, phthalocyanine acrylate as a lithium chelate monomer as the functional monomer, and EGDMA as the cross-linking agent. Characterization of the synthesized IIP using FTIR spectroscopy and SEM confirmed its suitable morphology and functional groups as an effective sorbent for SPE. Through the implementation of a central composite design (CCD) approach, the adsorption and desorption procedures were systematically optimized by varying the pH, temperature, sorbent mass, and elution volume. This systematic approach allowed for the identification of the most efficient operating conditions for extracting lithium from SWRO brine using IIP-SPE. Upon optimizing the conditions, the selectivity parameters demonstrated significantly enhanced affinity and selectivity for lithium in synthetic brines, particularly at high salt concentrations. The relative selectivity coefficients (α) against sodium and potassium were found to be 2.6 and 2.4, respectively, indicating a higher degree of preference for lithium adsorption. The adsorption data for Li on IIP showed an excellent fit to the Langmuir isotherm, with a maximum adsorption capacity of 3.2 mg g^−1^. During the study, the IIP was subjected to a desorption experiment on real samples from SWRO brine, which displayed a remarkable ion recovery percentage for Li adsorption. The efficiency of IIP regeneration was evaluated through a cycle of the adsorption–desorption process, which resulted in Li recoveries of up to 80%. Overall, this method shows great potential for selectively recovering lithium from reverse osmosis waste in desalination plants, thereby reducing the need for complex separation processes. The high market value of lithium, owing to its essential role in emerging technologies, makes this approach economically viable, environmentally friendly, attractive, and sustainable.

## Figures and Tables

**Table 1:** 4. IIP and NIP synthesis protocol for Li.

Polymer	Template	Function Monomer	Cross Linker	Mole Ratio Li:TMAPC:EGDMA	Progen	Initiator
IIP	Li	TMAPC	EGDMA	2:4:20	ACN	AIBN
NIP	-	TMAPC	EGDMA	4:20	ACN	AIBN

**Table 2:** Operational range of input variables for experimental design.

IIP Phase	Input Variables	Unit	Symbol	Levels	
				Lower	Upper
Adsorption Desorption	pH	-	x_1_	4.0	9.0
	Temperature	°C	x_2_	15.0	30.0
	Sorbent mass	mg	x_3_	1.0	2.5
	Elution solvent volume	mL	x_4_	3.0	6.0

**Table 3:** Analysis of physical and chemical properties of SWRO brine.

pH	Salinity (ppt)	TDS (g·L^−1^)	Conductivity (mS·cm^−1^)	Reference
7.85	48.15	52.34	83.62	Current study
7.89	61.7	67.64	91.56	[65]
8.0	*NR	58.85	*NR	[74]
7.0	*NR	69.17–72.36	88-132	[75]
9.0	NR	30.73	77	[76]

*NR: not reported.

**Table 4:** Central composite design matrix-observed response in adsorption step and desorption-observed response in adsorption step (retention %).

Run	X_1_	X_2_	X_3_	X_4_	Adsorption Efficiency (%) (Mean ± SD)	Extraction Efficiency (%) (Mean ± SD)
1	5	10	35	5	5.90 ± 0.41	70.02 ± 0.41
2	9	10	35	5	21.27 ± 0.73	65.15 ± 0.32
3	5	30	35	5	63.81 ± 0.34	72.21 ± 0.71
4	5	10	75	5	19.40 ± 0.67	76.31 ± 0.62
5	5	30	75	5	22.60 ± 1.13	64.22 ± 0.68
6	9	30	75	5	64.90 ± 0.82	78.21 ± 0.86
7	5	10	35	10	5.90 ± 0.46	81.31 ± 0.51
8	9	10	35	10	21.27 ± 0.36	84.52 ± 0.46
9	5	30	35	10	43.60 ± 0.67	87.12 ± 0.74
10	9	30	35	10	66.8 ± 0.44	88.41 ± 0.54
11	5	10	75	10	3.40 ± 0.19	89.12 ± 0.33
12	9	10	75	10	19.30 ± 0.56	96.67 ± 0.57
13	9	30	75	10	63.81 ± 0.81	98.62 ± 0.36
14	3.26416	20	55	7.5	7.30 ± 0.53	82.33 ± 0.48
15	7	38.6792	55	7.5	86.40 ± 0.64	92.18 ± 0.55
16	7	20	92.3584	7.5	21.40 ± 0.77	87.41 ± 0.68
17	7	20	55	12.1698	62.54 ± 0.48	97.56 ± 0.42
18	7	20	55	7.5	62.76 ± 0.66	96.21 ± 0.68

SD standard deviation for n = 3.

**Table 5:** Analysis of variance (ANOVA) for the response surface quadratic model (adsorption step).

Source	Sum of Squares	*df*	Mean Square	F-Ratio	*p*-Value
x_1_: pH	1194.43	1	1194.43	7.52	0.0712
x_2_: Sorbent mass	3933.06	1	3933.06	24.76	0.0156
x_3_: Temperature	115.585	1	115.585	0.73	0.4563
x_4_: Elution volume	105.372	1	105.372	0.66	0.4750
x_1_x_1_	965.825	1	965.825	6.08	0.0904
x_1_x_2_	153.986	1	153.986	0.97	0.3974
x_1_x_3_	45.6497	1	45.6497	0.29	0.6291
x_1_x_4_	3.16847	1	3.16847	0.02	0.8966
x_2_x_2_	192.25	1	192.25	1.21	0.3516
x_2_x_3_	341.464	1	341.464	2.15	0.2389
x_2_x_4_	1.46013	1	1.46013	0.01	0.9297
x_3_x_3_	908.493	1	908.493	5.72	0.0966
x_3_x_4_	4.23446	1	4.23446	0.03	0.8807
x_4_x_4_	0.0139282	1	0.0139282	0.00	0.9931
Total error	476.561	3	158.854		
Total (corr.)	12,418.0	17			
Multiple R^2^ = 0.961623; Adjusted R^2^ = 0.78.2533					
Standard Error of Est. = 12.6037; Mean absolute error = 4.44937					
Durbin–Watson statistic = 1.61883 (*p* = 0.1563)					

*df* is the degree of freedom.

**Table 6:** Analysis of variance (ANOVA) for the response surface quadratic model (desorption step).

Source	Sum of Squares	*df*	Mean Square	F-Ratio	*p*-Value
x_1_: pH	39.9236	1	39.9236	2.31	0.2261
x_2_: Sorbent mass	1.45796	1	1.45796	0.08	0.7905
x_3_: Elution flow rate	116.606	1	116.606	6.74	0.0807
x_4_: Elution volume	783.902	1	783.902	45.30	0.0067
x_1_x_1_	97.0165	1	97.0165	5.61	0.0987
x_1_x_2_	8.86332	1	8.86332	0.51	0.5259
x_1_x_3_	58.1122	1	58.1122	3.36	0.1642
x_1_x_4_	4.79741	1	4.79741	0.28	0.6350
x_2_x_2_	30.3584	1	30.3584	1.75	0.2772
x_2_x_3_	40.2063	1	40.2063	2.32	0.2248
x_2_x_4_	10.3525	1	10.3525	0.60	0.4956
x_3_x_3_	177.623	1	177.623	10.27	0.0492
x_3_x_4_	2.78512	1	2.78512	0.16	0.7151
x_4_x_4_	176.978	1	176.978	10.23	0.0494
Total error	51.9111	3	17.3037		
Total (corr.)	1984.44	17			
Multiple R^2^ = 0.9738; Adjusted R^2^ = 0.8517					
Standard Error of Est. = 4.15977; Mean absolute error = 1.4799					
Durbin–Watson statistic = 2.11177 (*p* = 0.4848)					

*df* is the degree of freedom.

**Table 7:** Selectivity factors of Li-imprinted polymers in synthetic brines for Lithium, compared to Na and K at temperature T = 45 °C and pH = 8.

Synthetic Brine	Selectivity Separation Factor	Selectivity Separation Factor
	α_Li/Na_	α_Li/K_
* Brine (I)	3.6	3.4
** Brine (II)	2.6	2.4

(*): Continuous flow using SPE columns; (**): Batch experiments. Brine 1 (low concentration): 50 mg/L Li^+^, 50 mg/L Na^+^, and 50 mg/L K^+^, Brine II (high concentration): 50 mg/L Li^+^, 30,000 mg/L Na^+^, and 1500 mg/L K^+^.

**Table 8:** Selectivity factors of Li-imprinted polymers in synthetic brines for Lithium, compared to Ca and Mg at temperature T = 45 °C and pH = 8.

Synthetic Brine	Selectivity Separation Factor	Selectivity Separation Factor
	α_Li/Ca_	α_Li/Mg_
Brine (III)	1.85	0.96

**Table 9:** Recovery of Li from spiked SWRO brine under optimal conditions.

Spiked SWRO Brine (mg·L^−1^)	Recovery (%)	RSD (%) (n = 3)
5	62.81 ± 5.38	5.68
10	66.38 ± 6.28	6.82
20	71.53 ± 4.25	4.57

RSD relative standard deviation.

## Data Availability

Not applicable.
